# Pattern of otitis externa in Kaduna Nigeria

**DOI:** 10.11604/pamj.2015.21.165.5577

**Published:** 2015-06-30

**Authors:** Thomas Samdi Musa, Alfred Nicholas Bemu, Umar Sambo Grema, Abdullahi Musa Kirfi

**Affiliations:** 1 Consultant ENT Surgeons, Department of Clinical Services, National Ear Care Centre, Kaduna, Nigeria; 2 Registrar ENT, Department of Clinical Services, National Ear Care Centre, Kaduna, Nigeria

**Keywords:** Pattern, otitis externa, Kaduna

## Abstract

**Introduction:**

Otitis externa (OE) is an inflammation or infection of the external auditory canal (EAC), the auricle, or both this condition has been reported to be found in all age groups. The aims and objectives were, study/determine the prevalence of Otitis externa in the specialist otolaryngology clinic in National Ear Care Center Kaduna, study the pattern of presentation among patients with otitis externa in the specialist otolaryngology clinic in National Ear Care Center Kaduna, and evaluate the choice of drug treatment for otitis externa in the specialist otolaryngology clinic in National Ear Care Center Kaduna.

**Methods:**

Data of patients diagnosed with otitis externa between January 2009 and March 2013 were extracted from the recorded cases of ear disease seen within the same period. The ages, sex/ gender, complains(symptoms), duration of symptoms, clinical examination findings, diagnosis, mode of drug treatment, number of visits and complication records were extracted from the case notes of the patients and analyzed descriptively using SPSS (Statistical package for Social Sciences) version.

**Results:**

Out of 13,328 cases of ear diseases seen within the period under review, 133 cases were diagnosed with otitis externa across all age groups. Hospital prevalence stands at 1.0%. There were 81(60.9%) males and 52(39.1%) females in ratio 1.5:1. Children age 0-15 constitute 55(41.3%) while young adults and adults were 78(58.6%). The minimum age at presentation was one year, while maximum age was 64 years. Mean age was 24 years with a standard deviation of ± 1.12 Years. Ear pain as only presenting symptom was the major complain found in this study accounting for 68(51.1%). Acute diffuse otitis externa was the commonest diagnosis accounting for 101(75.9%) and associated clinical findings ranging from tragal tenderness, hyperaemia and oedema of ear canal in 57 (54.9%). Ear swab was not routinely done and only 6(15.8%) of the discharging ears had microscopy done and the organisms were *Pseudomonas* spp and klebsiella. Empirical treatment was the commonest treatment modality and about 91% of the patients had complete symptom resolution by second visit. Complication was observed in only one case of necrotizing otitis externa who was retro-viral positive.

**Conclusion:**

Otitis externa accounted for small fraction of cases seen in our clinic (1%). Acute diffuse otitis externa is the commonest diagnosis made with symptoms ranging from ear pain, ear discharge, hearing loss and itchiness. Most patients were treated empirically with significant success within first two visits. No major complication was recorded within the period under study.

## Introduction

Otitis externa (OE) is an inflammation or infection of the external auditory canal (EAC), the auricle, or both [1]. This conditions have been reported to be found in all age groups [2]. Treatment of otitis externa is dependent on a thorough understanding of anatomy and physiology of the external ear canal, knowledge of the microbiology of potential pathogens, and familiarity with clinical presentation, so that an accurate and timely diagnosis can be reached [1]. It is a common disease condition affecting 5-20% of all patients attending otolaryngology clinic [3]. The aim of this study is to examine the pattern, clinical features and treatment response (as depicted by number of visits) of otitis externa at the study center.

## Methods

Data of patients diagnosed with otitis externa between January 2009 and March 2013 were extracted from the recorded cases of ear diseases seen within the same period. Demographic data (age, sex/gender), presenting symptoms and the duration, clinical examination findings, diagnosis, mode of drug treatment, number of visits and complication records were extracted from the case notes of the patients and analyzed descriptively using SPSS (Statistical package for Social Sciences) Version….

## Results

Out of 13,328 cases of ear diseases seen within the period under review, 133 cases were diagnosed with otitis externa across all age groups. Hospital prevalence stands at (1.0%). There were 81(60.9%) males and 52(39.1%) females in ratio 1.5:1. Children age 0-15 constitute 55(41.3%) while young adults and adults were 78(58.6%). The minimum age at presentation was one years while maximum age was 64 years. Mean age was 24. years with a standard deviation of ± 1.12 Years. Majority presented within two weeks of onset of symptoms 104(78.2%), while 21.8% presented within 3 weeks and above. Children aged 0-15 years constitute 55(41.3%) while young adults and adults were 78(58.6%) of all cases of otitis externa as shown in Table 1 on page 9. Bilateral ear involvement was noted in only 10(7.6%) and right or left ears were affected in 66(49.6%) and 57(42.9%) respectively. Ear pain as only presenting symptom was the major complain found in this study accounting for 68(51.1%) (Table 2). This is closely followed by ear pain and associated discharge seen in 35(26.3%) of the cases. Ear pain and itchiness was documented in 27(20.3%) while discharge, itchiness and pain in 2(1.5%). Vesicular ear lesions, ear pain were documented in 1(0.8%). As shown in Table 3 on page 11, acute diffuse otitis externa was the commonest diagnosis accounting for 101(75.9%) and 57(54.9%) cases had clinical findings ranging from tragal tenderness, hyperaemia and oedema of ear canal. Twenty-eight (27.45%) had in addition discharging ears. Ear wax and foreign bodies were documented in 9(8.8%) and 3(2.9%) respectively. There were 7(5.3%) cases of acute localized otitis externa (furunculosis). Chronic otitis externa and associated clinical findings of debris, scaly skin (dermatitis) was found in 20(15.4%). Few cases of malignant otitis externa, trauma to ear canal and herpes zoster oticus were also documented. Ear swab for microscopy culture and sensitivity in discharging ears 38(28.6%) was not routinely done. Only 6(15.8%) of the discharging ears had microscopy done and the organisms were Pseudomonas spp and klebsiella while 32(84.2) had none. The ears that were not discharging constitute 95(71.4%). Majority of cases 84 (63.2%) were treated with both topical and systemic drugs, while 38(28.6%) and 11(8.3%) were treated with only topical and systemic drugs respectively. No surgical procedure was required other than ear syringing and wig dressing in few cases. Follow-up record shows 121(91%) had complete symptom resolution by the second visit while 9(6.8%) third to fourth and 3(2.3%) 5th to 6th visits. There were no documented complications in 99.2% of the cases however 1(0.8%) case of retroviral infection with malignant otitis externa had intracranial collection.

**Table 1:** Age and sex distribution

AGE (YEARS)	SEX		TOTAL N (%)
	MALE	FEMALE	
0-15	39	16	55 (41.3)
16-30	13	19	32(24.1)
31-45	15	12	27(20.3)
46-60	12	5	17(12.8)
61-75	2	0	2(1.5)
>75	0	0	0(0)
**TOTAL N (%)**	**81 (60.9)**	**52 (39.1)**	**133 (100)**

**N=Number of patients, %= Percentage of patients**

**Table 2:** Diagnosis versus presenting symptoms

DIAGNOSIS	EAR PAIN	EAR PAIN DISCHARGE	EAR PAIN ITCHINESS	EAR PAIN DISCHARGE ITCHINESS	EAR ITCHINESS	EAR PAIN RASHES AND DISCHARGE
ACUTE DIFFUSED OTITIS EXTERNA	63	27	9	2	0	0
ACUTE LOCALISED OTITIS EXTERNA	3	4	0	0	0	0
CHRONIC OTITIS EXTERNA	1	0	1	0	18	0
NECROTISING OTITIS EXTERNA	0	2	0	0	0	0
HERPES ZOSTER OTICUS	0	0	0	0	0	2
TRAUMA	1	0	0	0	0	0
**TOTAL N (%)**	**68(51.1)**	**33(24.8)**	**10(7.51)**	**2(1.5)**	**18 (13.53)**	**2(1.5)**

**N= Number of patients, %= Percentage of patients**

**Table 3:** Diagnosis versus clinical examination finding

DIAGNOSIS	Impacted wax	Discharge oedema	Tragal tenderness debris	Hyperaemia Oedema tragal tenderness	Vesicles and oedema	Mass oedema discharge	Foreign body	Debris Scaly Ear canal	Total N (%)
**Acute diffused otitis externa**	9	28	8	53	0	0	3	0	101 (78.9)
**Acute localized otitis Externa**	0	4	0	3	0	0	0	0	7(5.3)
**Chronic otitis externa**	0	1	4	1	0	0	0	14	20(15.03)
**Malignant otitis Externa**	0	0	0	0	0	1	0	0	1(0.8)
**Herpes zoster oticus**	0	0	0	0	1	0	0	0	2(0.8)

**N= Number of patients; % = percentage of patients**

## Discussion

In our study otitis externa constitute only 1.0% of all otologic cases seen in our clinic within the period under review. This is different from the findings of Ayotunde et al [4] in which he recorded a prevalence of 4.3%. Ibekwe et al [5] reported that otitis externa was the commonest condition seen in Niger Delta of Nigeria where it constitutes 21.28% of ontological cases seen. Although the southern part of Nigeria is more humid and could account for the high prevalence, the general outpatient department of the study center treats uncomplicated ear diseases. This could reduce the number of cases eventually seen at the specialist otolaryngology clinic. In a study of by Rowland et al [2], in United Kingdom, referral of otitis externa to secondary care was uncommon (3%). Otitis externa was a common otologic emergency in a study by Afolabi et al [6] and it is one of the causes of ear ache. It can be genetically predetermined or influenced (narrow canal, extensive ear wax formation or inherited eczematous tendency); environmentally induced by heat, humidity and swimming; traumatic and self-induced match stick, hairgrip or cotton bud scratch with subsequent infection as all contributory factor in our study. The male to female ratio was 1.5:1 however some studies found female preponderance [2]. Children age 0-15 constitute 55(41.3%) while young adults and adults were 778(58.6%) of all cases of otitis externa. Similar finding was reported by David et al [7] in which he found peaks in cases of Otitis externa in persons 7- 12 years of age. Rowland et al [2] also reported diagnosis of otitis externa to be common in all age groups and, except in the elderly however, the sex preponderance in his study was female. Bilateral ear involvement was noted in only 10(7.6%) and right or left ears were affected in 66(49.6%) and 57(42.9%) respectively. A study of community pseudomonas infection in Beirut, Lebanon by Usamah H et al [8], show that they were mostly associated with otitis externa and the patients had either unilateral or bilateral otitis externa. Table 2 on page 10 show ear pain was the major complain found in this study accounting for 68(51.1%). This is closely followed by ear pain and associated discharge seen in 35(26.3%) of the cases. Ear pain and itchiness was documented in 27(20.3%) while discharge, itchiness and pain were the presenting symptoms in 2(1.5%) patients. Studies conducted by Paul et al [9] also found the common presentation to range from mild discomfort, itching, and minimal oedema to severe pain, complete canal obstruction, and involvement of the pinna and surrounding skin. Pain is the symptom that best correlates with the severity of disease [9].

Acute diffuse otitis was the commonest diagnosis made followed by chronic otitis externa and acute localized otitis externa. David et al in their study in United States found acute otitis externa to be much more common compared to chronic otitis externa [7]. Ear swab for microscopy culture and sensitivity in discharging ears 38(28.6%) was not routinely done. Only 6(15.8%) of the discharging ears had microscopy done and the organisms were Pseudomonas spp and klebsiella while 32(84.2) had none. The ears that were not discharging constituted 95(71.4%). Majority of cases 84 (63.2%) were treated with both topical and systemic drugs, while 38(28.6%) and 11(8.3%) were treated with only topical or systemic drugs respectively. No surgical procedure was required other than ear syringing and wig dressing in few cases. Medline systematic review of the effects of empirical and prophylactic treatments for otitis externa showed that oral antibiotics, specialist aural toilet, topical acetic acid drops or spray, topical aluminum acetate drops, topical antibacterial, topical antifungals, topical anti-infective agents, topical corticosteroids, and water exclusion were effective in managing otitis externa [10]. This is further buttressed by the findings in our study which shows 121(91%) had complete symptom resolution by the second visit on empirical treatment only while 9(6.8%) third to fourth and 3(2.3%) 5^th^ to 6^th^ visits. There were no documented complications in 99.2% of the cases however 1(0.8%) case of retroviral infection with malignant otitis externa had intracranial collection. Necrotizing otitis externa is defined by destruction of the temporal bone, usually in people with diabetes or in people who are immune compromised, and can be life threatening [11]. In conclusion otitis externa accounted for small fraction of ear cases seen in our clinic (1%). Acute diffuse otitis externa is the commonest diagnosis made with symptoms ranging from ear pain, ear discharge, hearing loss and itchiness. Most patients were treated empirically with significant success within first two visits. No major complication was recorded within the period under study.

## Conclusion

Otitis externa accounted for small fraction of cases seen in our clinic (1%). Acute diffuse otitis externa is the commonest diagnosis made with symptoms ranging from ear pain, ear discharge, hearing loss and itchiness. Most patients were treated empirically with significant success within first two visits. No major complication was recorded within the period under study.
